# Refining perioperative immunotherapy strategies in resectable NSCLC: surgical and functional perspectives

**DOI:** 10.1097/JS9.0000000000003218

**Published:** 2025-08-20

**Authors:** Man Sun, Dan Zang, Jun Chen

**Affiliations:** Department of Oncology, The Second Hospital of Dalian Medical University, Dalian, Liaoning, China

*Dear Editor,*

The AEGEAN phase 3 trial has demonstrated that adding perioperative durvalumab to platinum-based chemotherapy improves nodal downstaging and R0 resection rates in resectable non-small cell lung cancer (NSCLC)^[1,2]^. As immunotherapy extends into the perioperative setting, several clinical and translational questions warrant closer attention. This study complies with the TITAN 2025 guidelines for transparent and ethical reporting of AI-assisted research[3].

Although enhanced nodal clearance was observed (e.g., N2–N0 conversion: 47.3% vs. 40.2%), such metrics may not fully capture the durable immune remodeling induced by immune checkpoint blockade. The therapeutic benefit likely stems from clonal T-cell expansion, memory formation, and immune microenvironment reprogramming – features not reflected by pathology-based endpoints alone[4]. Future trials should incorporate dynamic immunologic biomarkers such as circulating tumor DNA (ctDNA) clearance, T-cell receptor (TCR) clonality, and spatial immune profiling to define treatment efficacy beyond morphological regression. These biomarkers may offer earlier and more biologically relevant indicators of long-term benefit.

Importantly, none of the patients in AEGEAN with radiographic progressive disease (PD) achieved a pathological complete response (pCR) or major pathological response (MPR). This highlights a clinical paradox: the need to distinguish true progression from immunotherapy-related flare or pseudoprogression. Relying solely on imaging may result in premature discontinuation of effective therapy or inappropriate surgical delays. Early on-treatment ctDNA dynamics have emerged as promising surrogates for biological response and could guide surgical triage based on immune activity rather than anatomical stability[5]. This paradigm shift toward “biological resectability” may enable more precise preoperative decision-making in the era of immunotherapy.

While AEGEAN reported no significant negative impact on surgical feasibility, nuanced intraoperative and postoperative consequences deserve further scrutiny. Neoadjuvant immunotherapy can lead to fibrosis and nodal adhesions, increasing the complexity of resection, yet such factors are often subjectively assessed. Standardized intraoperative scoring systems and conversion rates should be reported to capture real-world technical challenges[6]. Physiologically, although ventilatory parameters (FEV_1_, FVC) may improve after tumor regression, paradoxical declines in diffusing capacity (DLCO) have been observed[7]. This may reflect immune-mediated endothelial or alveolar injury not evident on imaging or spirometry. Thus, DLCO monitoring should be integrated into preoperative evaluation, particularly as trials increasingly include patients with stage N2 disease and diverse baseline functional status. Additionally, functional recovery remains an underexplored dimension. Patient-reported outcome measures (PROMs) such as dyspnea, fatigue, and emotional well-being offer valuable insights into post-treatment recovery but remain underutilized[8]. Their integration into trial endpoints could ensure that therapeutic success aligns with patient-centered goals. As perioperative immunotherapy strategies evolve, attention must shift beyond tumor regression to include physiological resilience and quality of life.

In summary, AEGEAN marks a pivotal step in redefining perioperative strategies for NSCLC. To realize its full potential, perioperative immunotherapy must evolve beyond feasibility, integrating surgical complexity, functional outcomes, and patient-centered measures. This correspondence identifies key translational gaps in AEGEAN and advocates for biomarker-guided assessment and standardized surgical endpoints to advance resectable NSCLC care.

## Data Availability

No datasets were generated or analyzed for this article. Data sharing is not applicable.

## Research registration unique identifying number (UIN)

Not required, as this study did not involve human participants.
